# Undergraduate medical education programme renewal: a longitudinal context, input, process and product evaluation study

**DOI:** 10.1007/s40037-015-0243-3

**Published:** 2016-01-28

**Authors:** Azim Mirzazadeh, Roghayeh Gandomkar, Sara Mortaz Hejri, Gholamreza Hassanzadeh, Hamid Emadi Koochak, Abolfazl Golestani, Ali Jafarian, Mohammad Jalili, Fatemeh Nayeri, Narges Saleh, Farhad Shahi, Seyed Hasan Emami Razavi

**Affiliations:** Medical Education Department, Tehran University of Medical Sciences, Tehran, Iran; Anatomy Department, Tehran University of Medical Sciences, Tehran, Iran; Infectious Diseases Department, Tehran University of Medical Sciences, Tehran, Iran; Biochemistry Department, Tehran University of Medical Sciences, Tehran, Iran; Surgery Department, Medical Education Department, Tehran University of Medical Sciences, Tehran, Iran; Emergency Medicine Department, Medical Education Department, Tehran University of Medical Sciences, Tehran, Iran; Maternal Fetal and Neonatal Research Center, Tehran University of Medical Sciences, Tehran, Iran; Education Development Office, Tehran University of Medical Sciences, Tehran, Iran; Internal Medicine Department, Tehran University of Medical Sciences, Tehran, Iran

**Keywords:** Undergraduate Medical Education programme, CIPP model, Curriculum reform

## Abstract

The purpose of this study was to utilize the Context, Input, Process and Product (CIPP) evaluation model as a comprehensive framework to guide initiating, planning, implementing and evaluating a revised undergraduate medical education programme. The eight-year longitudinal evaluation study consisted of four phases compatible with the four components of the CIPP model. In the first phase, we explored the strengths and weaknesses of the traditional programme as well as contextual needs, assets, and resources. For the second phase, we proposed a model for the programme considering contextual features. During the process phase, we provided formative information for revisions and adjustments. Finally, in the fourth phase, we evaluated the outcomes of the new undergraduate medical education programme in the basic sciences phase. Information was collected from different sources such as medical students, faculty members, administrators, and graduates, using various qualitative and quantitative methods including focus groups, questionnaires, and performance measures. The CIPP model has the potential to guide policy makers to systematically collect evaluation data and to manage stakeholders’ reactions at each stage of the reform in order to make informed decisions. However, the model may result in evaluation burden and fail to address some unplanned evaluation questions.

## Essentials

- The CIPP model addresses all the steps of an education programme, even when the programme is still being developed.
- Context evaluation is very important in convincing the faculty members and policymakers at the onset of a major programme reform.
- Input evaluation might be helpful in saving precious resources which might be lost by performing evaluation at the end of the programme.
- The CIPP model provides ongoing information to decision-makers to ensure that the implemented programme is on the track.
- The CIPP evaluation model may fail to address some important but unplanned evaluation questions and may result in evaluation burden.

## Introduction

The past two decades have witnessed an international call for fundamental changes in medical education programmes [1‒3]. Many medical schools around the world initiated a new undergraduate medical education curriculum to meet the current demands of practice in medicine [4‒8]. The successful renewal in an undergraduate medical education programme requires that programme developers identify the needs of the learners and the community, plan a programme that can successfully address both sets of needs, and implement the programme in a way that satisfies the outcomes of the planned programme [9]. Hence, planners need a suitable model for generating helpful information and guiding them throughout the process of revision in the undergraduate medical education programme [10].

The use of the Context, Input, Process and Product (CIPP) evaluation model has been thoroughly recognized in a variety of educational and non-educational evaluation settings [11‒13]. Additionally, a number of studies that used this model to evaluate educational programmes in the context of health professions have attracted attention in the literature in recent years [14, 15]. However, to date, no comprehensive longitudinal study has used the CIPP evaluation model to facilitate informed decision-making in all stages of reform in an undergraduate medical education programme.

The CIPP evaluation model addresses all phases of an education programme renewal [16], accommodates the complex nature of medical education programmes, and provides formative information to stakeholders for the purpose of improvement and informed decision-making [17]. The first component, context evaluation, is useful when an established programme is going through a planned change or must adjust to changed conditions. The second component, input evaluation, helps to determine an appropriate programme model to satisfy the identified needs. Process evaluation provides formative information for guiding revisions and adjustments whilst the planned programme is running. The last component, product evaluation, produces valuable information in order to judge programme outcomes [18].

This article elaborated the use of the CIPP evaluation model as a comprehensive framework to help to initiate, develop, install, and evaluate a new undergraduate medical education programme in a period of eight years. We examined five specific research questions:

1. How does the CIPP evaluation model effectively facilitate the management of the stakeholders’ reactions during the undergraduate medical education programme reform?
2. What are the needs of the undergraduate medical students and the community?
3. What is an appropriate model for an undergraduate medical education programme to address the identified needs?
4. What are the strengths and weaknesses of the new undergraduate medical education programme?
5. To what extent has the new undergraduate medical education programme achieved its outcomes in basic science phases?

## Methods

### Study context

This study was conducted at the School of Medicine of Tehran University of Medical Sciences. This school, which is one of the largest and oldest among Iranian medical schools, delivered a traditional Flexnerian undergraduate medical education programme for a long period of time. This programme was composed of two and half years of basic sciences, one year of pathophysiology, a two-year clerkship, and an 18-month internship. The idea of reform in the traditional programme was raised seriously in the early 2000s when the change seemed inevitable in our institution in response to profound contextual changes and the recommendations of the Iranian Ministry of Health and Medical Education.

### Procedure

**Fig. 1:**
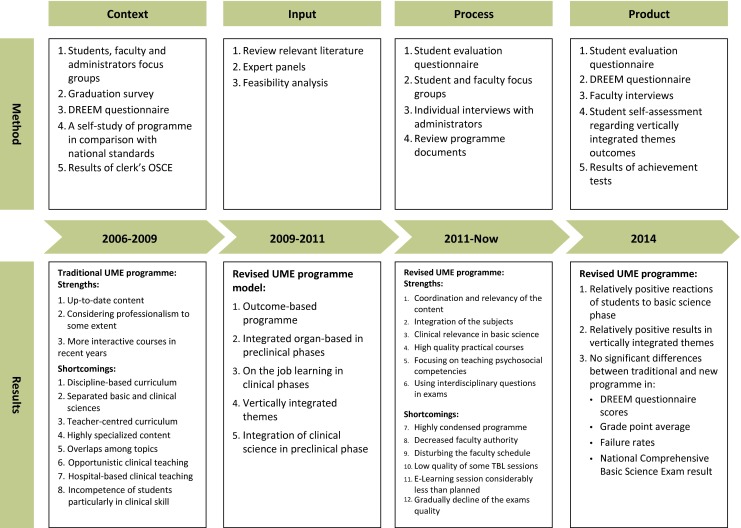
CIPP evaluation model. *OSCE* objective structured clinical examination, *TBL* team-based learning, *UME* undergraduate medical education

This longitudinal evaluation project started from 2006. The entire process was supervised by Educational Development Office of the School of Medicine. Fig. 1 depicts four complementary sets of evaluation phases which were compatible with the four components of the CIPP evaluation model. Phase 1 and 2 were conducted when the traditional undergraduate medical education programme was running while phase 3 started simultaneously with the onset of the renewed undergraduate medical education programme in 2011. Phase 4 was carried out three years after beginning the new programme. The Research Ethics Committee of Tehran University of Medical Sciences granted ethical approval for the study (IR.TUMS.REC.1394.801).

#### Phase 1: Context evaluation

In order to understand the necessity and scope of the change, we conducted a comprehensive context evaluation from 2006 to 2009 which comprised five projects. The projects included exploring the challenges of the traditional programme from the stakeholders’ viewpoint, evaluating the quality of the traditional programme in graduates’ perceptions, assessing the educational environment from the students’ perspectives using the Dundee Ready Education Environment Measure (DREEM) inventory, self-study of the traditional programme in comparison with the national undergraduate medical education standards and evaluating the competency of medical students in clinical skills through an objective structured clinical examination (Table 1).

**Table 1:** Context evaluation: methods and results

Method	Details of the method	Main results
1. Students, faculty and administrator focus groups	To explore the challenges of the traditional programme, 21 focus group sessions (12 with students, 7 with faculty and 2 with administrators) were conducted during May to July 2006. Each session were lasted 120–150 min. Data were analyzed using qualitative content analysis method	Four categories of challenges have been identified: 1. Challenges of the structure of the programme 2. Challenges of the content of the curriculum 3. Limitations of the resources 4. Challenges of the programme implementation
2. Graduation survey	To evaluate the perceptions of our graduates regarding the quality of the traditional programme, a 262-item questionnaire was developed based on the graduation survey by the Association of American Medical Colleges. A total of 183 questionnaires were completed by medical students upon their graduation from the medical school in 2007	- Satisfied with the medical training they received (28.4 %) - Basic science courses lacked clinical relevance (77 %) - Acquired adequate knowledge and skills to start residency training (33.3 %) - Have not been taught sufficient clinical skills in preparations for their future practice (70 %)
3. DREEM questionnaire	To evaluate the educational environments from perspectives of the students, a total of 541 students (103 basic sciences, 103 preclinical and 335 clinical students) completed the standard DREEM questionnaire in 2008	Overall DREEM questionnaire score was 91.46/200 (students’ perception of teaching [23.75/48], students’ perception of teachers [19.42/44], students’ academic self-perceptions [13.21/32], students’ perceptions of atmosphere [23.35/48], students’ social self-perceptions [13.99/28])
4. A self-study of programme in comparison with national undergraduate medical education standards	A self-study of the traditional programme was conducted on the basis of the national standards (including 9 domains and 57 standards) in 2007. 234 questionnaires were completed by students, faculty and administrators. 82 department deans, course directors and faculty used the results to evaluate the programme quality in comparison with each national standard on a scale from 0 to 100	Final results showed that 22 (40 %) standards were rated as ‘relatively match’ (50–75) and ‘completely match’ (75–100) by more than 50 % of the members of the workshops. 32 (55 %) standards were rated as ‘does not match’ (0–25) and ‘slightly match’ (25–50) by more than 50 % of the members of the workshops
5. Results of OSCE	86 students participated in an OSCE exam at the end of the clerkship period. The exam was conducted in the morning (2 different tracts) and in the evening (2 similar tracts). Each tract consisted of seven stations	There was a significant difference (*p* < 0.001) between history taking (accounted for the highest points) and procedure (accounted for the lowest points) stations

#### Phase 2: Input evaluation

In a two-year input evaluation project, we carried out three consecutive activities. In order to set down a sound model for the revised undergraduate medical education programme, the responsible taskforce reviewed the relevant literature on authoritative medical education journals and visited the websites of leading medical schools around the world and also collected national documents of undergraduate medical education programmes. Next, expert panels were held to generate a preliminary draft of the framework of the undergraduate medical education programme on the basis of context evaluation and literature review results. The preliminary draft was converted to the final version of the programme during a participatory process. Meetings were conducted with faculty members from different departments, medical school administration and students’ representatives in both basic and clinical sciences phases, as well as with the recent graduates, to receive their input. Finally, expert judgment was considered to determine the feasibility of the proposed model and adjustments were made to improve it. Overall, 170 faculty members and administrators participated in the input evaluation phase. We also asked three experts for their comments from abroad. We involved students considerably during the planning phase: 18 students in committees and subcommittees, 35 students in panels and some others in workshops.

#### Phase 3: Process evaluation

In September 2011, School of Medicine implemented the revised undergraduate medical education programme with extensive changes on the basis of input evaluation results. The process evaluation started from scratch when the new programme was launched. Information was regularly collected through diverse methods. For instance, online questionnaires were administered and focus groups were conducted after each interdisciplinary organ-system block in order to receive the students’ viewpoints. We also reviewed the course syllabi to make sure all classes and sessions were held as planned (Table 2).

**Table 2:** Process evaluation: methods and results

Method	Details of the method	Main results
1. Student evaluation questionnaire	An online, 40-item questionnaire on a 5-point Likert scale (ranging from 1 = strongly disagree to 5 = strongly agree) was completed by students at the end of each interdisciplinary block regarding the quality of the blocks. A total of 1004 questionnaires were completed for 10 blocks. Mean response rate for each block was 63 %	Most students agreed or strongly agreed that: - Block material was appropriate (77.7 %) - Block content was related to and consistent with each other (77.4 %) - Integrated content contributed significantly to their learning (73.6 %) - Interdisciplinary questions were suitable (72.4 %) More students disagreed or strongly disagreed that: - Enough time was allotted for subjects (56.9 %) - Block instructors used techniques like questioning to make sessions interactive (47.7 %)
2. Student and faculty focus groups	To identify strengths and shortcomings of the implemented revised programme, 15 focus group sessions were conducted during December 2011 to December 2014 (12 sessions with students and three sessions with basic science faculty). Each session lasted 30–90 min which were audiotaped and transcribed	Strengths: - Integration of basic science subjects - Case-based discussion sessions Shortcomings: - Insufficient coordination among the block teachers - Low quality of some team-based learning sessions - Low quality of some exam questions - Disturbing the faculty schedule - Uncertainty about the success of the programme (at the early stage)
3. Individuals interviews with administrator Review the programme documents	To identify the extent to which the revised programme was implemented as planned, interviews were conducted with six reform committee chairs. Course syllabi and exam questions were reviewed as well	Holding lectures and practical sessions as planned - E-learning session considerably less than planned - Gradual decline of the exams quality

#### Phase 4: Product evaluation

The main purpose of the fourth component, product evaluation, was to ascertain the extent to which the targeted educational needs were met. Although the revised programme has a rather long way to go to attain its long-term outcomes, we have already examined the outcomes of the basic sciences phase. We investigated the reaction of students and faculty members to the programme as well as the quality of student learning after completion of the basic sciences phase (Table 3).

**Table 3:** Product evaluation: methods and results

Method	Details of the method	Main results
1. Student evaluation questionnaire	To evaluate the students’ perceptions regarding the quality of the basic science phase and its application to the next phase, a 96-item questionnaire was developed based on the graduation survey by the Association of American Medical Colleges. A total of 136 students (response rate, 51 %) completed the questionnaire four month after completing the basic science phase in June 2014	More students agreed or strongly agreed that: - Overall, I am satisfied with the quality of my basic science (53.7 %) More students disagreed or strongly disagreed that: - Basic science content had sufficient illustrations of clinical relevance (50.7 %)
2. Student self-assessment regarding vertically integrated themes outcomes	23 items of the above-mentioned questionnaire were related to outcomes of the vertically integrated themes	More students agreed or strongly agreed that: - In practical classes, my behaviour and appearance are appropriate to the medical profession (78.7 %) - I do not hesitate to share my knowledge and ability to my classmate during the group work (77.2 %)
3. DREEM questionnaire	A total of 102 students (response rate, 44 %) enrolled in 2011 and 197 students (response rate, 87 %) enrolled in 2010 completed the standard DREEM questionnaire after completing their basic science phase	No significant differences were found between traditional and revised programme in Overall DREEM questionnaire scores. Students in revised programme evaluated the educational environments in 10 items significantly better than students in traditional programme (items 2, 5, 9, 11, 16, 28, 30, 37, 39 and 44)
4. Individuals interviews with faculty	Individual interviews were conducted with 14 basic science faculty 3 years after running the revised programme. Each session lasted 20–45 min	Faculty concerns were: - Decreased faculty authority - Disturbed faculty schedule - Gradual weakening of the programme - Influence on student learning - Insufficiency of team-based learning
5. Results of achievement tests	To compare the student performance in exams in the revised and traditional programme, a total of 724 exams results were extracted related to the: - 231 students of the revised programme enrolled in 2011 - 225, 184 and 184 students of the traditional programme enrolled in 2010, 2009 and 2008, respectively	No significant differences (*p*_s_ > 0.05) were detected between traditional and new programme in: Grade point average Failure rates National Comprehensive Basic Science Exam result

### Strategies for managing stakeholders’ reactions

In addition to applying four components of the CIPP evaluation model, we benefited from other features of the model, as follows, to manage the stakeholders’ reactions during the undergraduate medical education programme reform.

#### Triangulation of evaluation sources and methods

The CIPP evaluation model allows evaluators to apply several data collection and analysis methods necessary for triangulation of data and in turn increases the validity of the evaluation results [19]. We collected evaluation data from different sources such as medical students, faculty members, administrators and graduates using various qualitative and quantitative methods such as focus groups, questionnaires and performance measures during all phases and within each phase to address evaluation questions properly.

#### Stakeholders’ involvement in the evaluation process

The CIPP evaluation model places the emphasis on the engagement of stakeholders in the evaluation process [18]. The strategies that we employed to involve our stakeholders in all stages of the reform process included: setting up different committees and subcommittees with participation of faculty members from different departments and representatives of students; communicating the reform plan and progress with stakeholders through written evaluation reports, meetings, seminars, websites, handbooks, newsletters and so on; reducing the central management of the programme and giving more responsibilities to block directors after establishment of the programme; involving faculty members in proposing revision strategies during the process phase and finally holding various faculty development courses.

#### Ongoing formative evaluation

The most important purpose of the CIPP evaluation model is improvement [10]. We established an internal evaluation system, under the responsibility of the evaluation committee of the undergraduate medical education programme, to monitor and improve the new programme through its installation.

## Results

### Phase 1: Context evaluation

The results of the context evaluation revealed the current status of the traditional programme and expectations of a revised programme [20, 21]. Excess emphasis on highly specialized biomedical knowledge without paying attention to psychosocial aspects of care, teacher-centred curriculum and opportunistic clinical teaching were the most prominent weaknesses of the traditional programme (Table 1). This first step of evaluation led to identification of the reform priorities including implementing horizontal and vertical integration in the curriculum, improving student-centred learning, teaching in primary health care services, addressing the psychosocial aspects of patient care and applying authentic student assessment methods.

### Phase 2: Input evaluation

Input evaluation resulted in designing a model for a new undergraduate medical education programme which was mainly outcome-based. The graduates’ competencies were developed in line with the local situation [22, 23]. While the major change in the preclinical phases was developing integrated organ-based blocks, the most significant modification in the clinical phases was on the job learning. Furthermore, developing vertically integrated themes focusing on ethics, professionalism, communication skills, critical thinking, and comprehensive care was another significant revision in the new programme. We limited vertical integration to the early clinical exposure module, some case-based discussion sessions, and clinical application examples during the lecture throughout the basic sciences phase. We chose team-based learning [24] from a range of student-centred teaching methods identified in the literature [25] for the basic sciences phase. In addition, designing multidisciplinary questions and calculating cumulative disciplinary scores were among the changes made in the field of student assessment ([26]; Fig. 1).

### Phase 3: Process evaluation

Process evaluation identified the strengths and weaknesses of the revised programme as soon as it was launched. For instance, the students valued integration in basic science disciplines, clinical case discussion sessions, and interdisciplinary exam questions. They had concerns about the quality of team-based learning sessions and some of the questions in the block exams. Faculty was concerned about the quality of some elements of the programme, student and faculty overload as well as the disturbance of their schedules in the new programme (Table 2).

### Phase 4: Product evaluation

Product evaluation revealed the extent to which the new programme achieved its outcome for the basic science phase. For example, 53.7 % of the students were very satisfied or satisfied (in a five-point Likert scale from very dissatisfied to very satisfied) with the basic science phase, and 56.12 % of the students strongly agreed or agreed (in a five-point Likert scale from strongly disagree to strongly agree) that they achieved the vertically integrated theme outcomes. The DREEM questionnaire scores, student grade point averages, the failure rates, and National Comprehensive Basic Science Exam results did not differ significantly between the traditional and renewed curriculum (all *P* > 0.05) (Table 3).

### Successes and challenges in managing stakeholders’ reactions

By employing unique features of the CIPP evaluation model, we succeeded in convincing stakeholders of the need for major changes in the undergraduate medical education programme. We were also successful in creating a sense of ownership of the new programme in our stakeholders. We achieved some success in reassuring students, faculty members and administrators during the programme installation about the programme progress by continuous process evaluation and initial product evaluation, communicating successes and challenges with the stakeholders and using evaluation results for improvement. However, proving the effectiveness of the programme demands the passage of time. We also found that maintaining stakeholders’ collaboration and enthusiasm was a difficult task. Hence, establishing a reward system for compensating faculty participation and overload teaching might be beneficial.

## Discussion

The aim of this evaluation study was to utilize the CIPP evaluation model to assist decision-makers to initiate, develop, establish, and evaluate a revised undergraduate medical education programme in one medical school in Iran. This is the first study applying all four interrelated components of the CIPP evaluation model in a longitudinal work throughout the renewal cycle of an undergraduate medical education programme. The results of this study showed that the components of the CIPP evaluation model could successfully address all steps of the reform even when the new programme is still being developed. We took advantage of context and input evaluation before, as well as process and product evaluation after the implementation of the new programme. Context evaluation identified the weaknesses and strengths of the traditional programme and the needs of the learners and the community which, in turn, directed the rest of the renewal process. Input evaluation resulted in formulating a new programme tailored to our context and was helpful in saving precious resources. Process evaluation enabled us to improve the weaknesses early on in the reform installation. Product evaluation examined the extent of the initial achievements in the outcomes in basic science phase.

Few studies have applied the CIPP evaluation model longitudinally to the evaluation of medical education programmes. Steinert et al. [14] conducted all four elements of the CIPP evaluation model from the initial steps of the planning to implementation and evaluation of a faculty development programme aimed to promote the teaching of professionalism to medical trainees. Although their results were promising, more work is needed to examine the utility of elements of the CIPP evaluation model through the continuum of medical education programmes.

The CIPP evaluation model was also helpful in managing the stakeholders’ reactions through the reform process. We found it challenging to initiate and sustain major reforms in the undergraduate medical education programme in a large and old medical school with a history of success. Therefore, we conducted a comprehensive context evaluation with triangulation of the evaluation sources and methods. The context evaluation revealed the problems of the traditional programme deeply and broadly, which was very helpful to convince decision-makers and faculty members about the need for broad changes in the programme. Triangulating the evaluation data has been mentioned in the literature on medical education reform as an important factor to create the need for change as well [27, 28].

Once the need for change was confirmed, the prevailing reaction of the stakeholders was that our medical school is different and models of the undergraduate medical education programme reform are not necessarily suitable to our context. The steps taken during the input evaluation phase along with extensive stakeholder engagement were effective strategies to design an educationally sound undergraduate medical education programme that was readily adaptable to our situation and more importantly was accepted by the programme stakeholders [29].

Ongoing process evaluation and formative product evaluation with the use of qualitative methods enabled us to explore stakeholders’ reactions systematically during the implementation of the revised programme. For example, the first cohort of students was confused about the details of the reform and concerned about the success and continuation of the programme (Table 2). Additionally, some faculty members complained about the decrease in their authority and gradual decline of the programme quality (Tables 2 and 3). Regular reporting of the evaluation results and subsequent improvement were among the reassuring strategies that we employed. However, sustained efforts are required until the revised programme is fully established. Our experience was consistent with the history of change in medical education curricula that emphasizes the role of ongoing programme evaluation, use of qualitative data and communicating the evaluation results with stakeholders as essential elements to successful reform [30].

Applying the CIPP evaluation model is a time-consuming and demanding task which needs full administrative support and leadership stability. We faced the challenge of four changes in medical school dean during the reform process. However, the behaviour of all the administrators was supportive and we dealt with this instability successfully by creating a shared responsibility for the reform process between different groups of stakeholders. We also found that gathering evaluation data from different sources, and combining and timely reporting of these triangulated data, were difficult tasks that needed administrative support and expertise. We tried to overcome these challenges by involving our medical students in graduate courses in the evaluation process. We also assigned some parts of the evaluation practice to a volunteered group of medical students. We intend to involve the course directors in gathering evaluation data directly from students in the near future. Although these strategies were helpful, medical schools that may consider using this model should prioritize the evaluation questions carefully in order to manage the evaluation burden. Another weakness of the CIPP evaluation model was its focus on evaluating the predetermined plan and product. Therefore, some important questions such as the extent and nature of unintended outcomes of the programme might have remained unanswered in our study. Beyond the limitations of the CIPP evaluation model, our study mainly focused on the basic sciences phase of the undergraduate medical education programme during the process and product evaluations. We need to continue the project to examine the extent of outcome achievement, especially in the clinical phase.

The CIPP evaluation model has the potential to guide policy makers and other stakeholders to systematically collect evaluation data at each stage of reform in an undergraduate medical education programme in order to make informed decisions. Moreover, this model seems useful in managing the change process in terms of stakeholders’ reactions. The use of this evaluation model in other programmes in the context of medical education should be further studied.
